# Editorial: The evidence and practice-gap of screening and brief interventions for substance misuse

**DOI:** 10.3389/fpsyt.2022.1056814

**Published:** 2022-11-11

**Authors:** Abhishek Ghosh, Surendra K. Mattoo, Dorothy Newbury-Birch

**Affiliations:** ^1^Department of Psychiatry, Drug Deaddiction and Treatment Center, Postgraduate Institute of Medical Education and Research, Chandigarh, India; ^2^Adesh Medical College, Kurukshetra, Haryana, India; ^3^Centre for Social Innovation, Alcohol and Public Health Research, Teesside University, Middlesbrough, United Kingdom

**Keywords:** brief intervention (BI), alcohol, drugs, implementation, research

Screening and brief intervention (SBI) is a low-intensity, time-limited psychosocial treatment for substance misuse. The therapeutic principle is grounded on the social cognitive theory encompassing different combinations of personalized feedback, styles of motivational interviewing (MI), decisional balance, advice, identifying and managing high-risk situations, and providing a personalized menu of options to reduce or stop substance use (1). SBI could be limited to brief structured advice or extended as an MI-based intervention with more than one session and can be as little as 5 min (2). Heather, one of the architects of SBI, has termed it a “family of interventions” (3). The essence of SBI lies in its flexibility- different delivery settings, delivery by a wide range of professionals or through mobile or internet, and effectiveness across age groups. Modeling studies show alcohol BI is cost-effective in terms of the quality-adjusted life year gains and provides modest cost savings to the healthcare system (4, 5). Although during the first couple of decades, SBI was tested for problem alcohol use, in the last two decades, it has been tested for drug misuse and problem behaviors (6). The brevity, flexibility, and cross-cutting application have made SBI scalable, even in resource-limited settings. Therefore, SBI could become a public health tool to reduce the substantial disability attributed to substance use (7). SBI, in conjunction with other population-level measures for alcohol use, can help realize the global non-communicable disease target of a 10% reduction in harmful alcohol use. Despite being a promising intervention, SBI has several evidence and implementation gaps that must be addressed.

Although there are several screening instruments to detect and monitor substance misuse, the validity of those screening tools across countries, age groups, and study settings are not yet consistent (8). For instance, the Alcohol Use Disorder Identification Test (AUDIT), which could be the most widely used screener for alcohol misuse, requires adaptation in the first three consumption questions according to the country-specific definition of standard drinks and guidelines for low-risk drinking; however, this is seldom practiced (9). The detection rate might be better for alcohol than drug misuse; screening at any visit by a self-administered questionnaire might yield better detection rates than routine annual screening and administration by clinical staff (10). However, more research from different cultures and contexts is needed to find a valid and reliable screening tool to detect at-risk substance users and the optimal time and mode of administration.

The effectiveness of alcohol BI among adults in primary and general healthcare is consistent and robust (11, 12). However, the evidence for alcohol BI is limited among younger and older people and special populations or contexts or other settings such as the criminal justice system and general medical settings (13). There is a relative paucity of research from low-middle-income countries (LMIC). Nevertheless, a synthesis of the existing research shows a modest effect of BI in the adult population with alcohol misuse (14). Evidence of efficacy for BI is limited, at best mixed for unhealthy drug use (15, 16). The inconsistent results might include the heterogeneity in the study population, the presence of concomitant alcohol misuse or mental health problem, and the potential for under-reporting because of the criminal sanctions against drugs (17). Although there are validated screening instruments for substance misuse for the adult primary care population, we need further research on the screening instruments for unhealthy drug use and alcohol use problems among adolescents (8). Another vexing problem in SBI research is the inconsistency and lack of standardization of intervention outcomes. Hence, synthesizing results of clinical trials in meta-analyses becomes difficult or lacks sufficient power for a meaningful conclusion. Recently special interest group INEBRIA (International Network on Brief Interventions for Alcohol and Other Drugs) published a core set of outcomes for alcohol BI trials (18). Standardization of outcomes will improve reporting, aid comparability of studies, and help drive policy decisions. We need a similar core set of outcomes for drug misuse and BI trials conducted in other contexts, such as the workplace, criminal justice system, schools, or other academic institutions.

The implementation gap of BI is possibly much wider than the evidence gap, especially for alcohol misuse. The practice of alcohol BI in clinical settings, despite the support from policymakers, is sub-optimal. Scandinavian countries, the UK, and Italy, which have integrated alcohol BI into their healthcare system, show only a minority population are screened (10–37%) for alcohol use by their primary care physicians, and a further minority receive advice to cut down (19). The possible reasons for the implementation gap might include resource paucity (e.g., time and human resources), limited skill for delivering BI, and a perceived lack of capability or effectiveness of BI (20). A five-country European and UK study shows that financial incentives and training support improve alcohol screening in primary care (21). However, an observational study of English primary care finds no increase in alcohol screening and intervention following the introduction of financial incentives; nevertheless, withdrawal of the incentive is associated with a declining rate of screening and advice (22). The conflicting results between the trial and the naturalistic study might raise doubts about the real-world effectiveness of financial incentives to reduce the implementation gap. Digital SBI has the potential to improve access and reduce the implementation gap; however, digital SBI is grappling with some major challenges, such as sustainability, competing interests of for-profit entities, and unvalidated and non-evidence-based content (23). For a population-level impact, more than 90 percent of persons in primary care, irrespective of their reasons for consultations, should be screened for alcohol use, and at least 70% should receive brief advice (24). The implementation gap and lack of consistent evidence of measures that can improve this gap are major impediments to realizing the public health impact of alcohol BI.

This Research Topic revisits the evidence of alcohol BI in the criminal justice system and the integration of alcohol intervention into general healthcare settings. The latter study focuses exclusively on sub-Saharan Africa. These two studies aim to review and update the evidence of alcohol BI in non-primary care and low-resource settings. Levy et al. examined the uptake and preliminary outcomes of a teleconsultation model for pediatric primary care. The teleconsultation program from Massachusetts, US, provides an implementation model for SBI in children and adolescents in a limited resource context. Another electronic health record system-based study from the USA tested the changes in unhealthy alcohol use pre- and post-pandemic. The authors identified vulnerable subgroups and suggested using telehealth to reach out to these groups. Al Mahmud et al. explored the format and themes of another digital self-help option for alcohol cessation, the YouTube videos. In another study, the authors explored the need for brief counseling and intervention based on a theory-driven approach in the context of sexualized drug use, also known as Chemsex. Future studies on BI in special populations and contexts might use a theory-driven, tailor-made approach (rather than the common social cognitive approach). Finally, a South African study proposes screening for suicidal behavior in persons with heroin misuse- increasing the ambit of screening in SBI.

This Research Topic highlights the evidence and the implementation gap of SBI in substance misuse. This unpacking of the research gaps may motivate further research on SBI. It may provide directions to funders and policymakers.

## Author contributions

AG, SM, and DN-B jointly conceived the idea of the Research Topic and the editorial. AG wrote the first draft. SM and DN-B reviewed and edited the draft. All authors reviewed the final draft and approved it.

## Conflict of interest

The authors declare that the research was conducted in the absence of any commercial or financial relationships that could be construed as a potential conflict of interest.

## Publisher's note

All claims expressed in this article are solely those of the authors and do not necessarily represent those of their affiliated organizations, or those of the publisher, the editors and the reviewers. Any product that may be evaluated in this article, or claim that may be made by its manufacturer, is not guaranteed or endorsed by the publisher.
